# Iron Availability Modulates the Persistence of *Legionella pneumophila* in Complex Biofilms

**DOI:** 10.1264/jsme2.ME16010

**Published:** 2016-09-14

**Authors:** Emilie Portier, Joanne Bertaux, Jérôme Labanowski, Yann Hechard

**Affiliations:** 1 University of Poitiers, Laboratory of Ecology and Biology of InteractionsUMR CNRS 7267, Team of Microbiology of Water, PoitiersFrance; 2 University of Poitiers, Laboratory of Ecology and Biology of InteractionsUMR CNRS 7267, Team of Ecology Evolution and Symbiosis, PoitiersFrance; 3 University of PoitiersUMR CNRS 7285, Institute of Chemistry of Materials and Environments of PoitiersFrance

**Keywords:** *Legionella pneumophila*, biofilm, iron, chelator, river water

## Abstract

*Legionella pneumophila* is a pathogenic bacteria found in biofilms in freshwater. Iron is an essential nutrient for *L. pneumophila* growth. In this study, complex biofilms were developed using river water spiked with *L. pneumophila*, and the persistence of *L. pneumophila* in these complex biofilms was evaluated. In order to study the role of iron in the persistence of *L. pneumophila*, river water was supplied with either iron pyrophosphate or iron chelators (deferoxamine mesylate, DFX for ferric iron and dipyridyl, DIP for ferrous iron) to modulate iron availability. The addition of iron pyrophosphate and DFX did not markedly affect the persistence of *L. pneumophila* in the biofilms, whereas that of DIP had a beneficial effect. Since DIP specifically chelates ferrous iron, we hypothesized that DIP may protect *L. pneumophila* from the deleterious effects of ferrous iron. In conclusion, ferrous iron appears to be important for the persistence of *L. pneumophila* in complex biofilms. However, further studies are needed in order to obtain a better understanding of the role of ferrous iron in the behavior of this bacterium in the environment.

*Legionella pneumophila* is a waterborne bacterium that is responsible for Legionnaire’s disease (12, 15, 27). This bacterium is transmitted to man by the inhalation of contaminated aerosols and has the ability to grow within the lungs because of its potential resistance to macrophage phagocytosis (11, 18).

In freshwater environments, this pathogen is mainly found as sessile cells associated with biofilms (7–9, 21, 40). Biofilms are microbial sessile communities attached to a substratum embedded in a secreted extracellular matrix and exhibit a specific phenotype (10). Within the biofilm, the main site of multiplication is likely inside protozoan hosts, such as free-living amoebae (20, 30). *L. pneumophila* is able to resist amoeba phagocytosis and even multiplies within amoebae (37).

Due to the complexity of biofilms that develop in natural environments, the behavior of *L. pneumophila* has mainly been studied in mono- or mixed species biofilms (17, 26, 31, 32, 39). These biofilms are convenient for mechanistic studies, but do not reflect environmental biofilms. *L. pneumophila* may represent a minor species in environmental biofilms, and, as such, other microorganisms may have an impact on the occurrence of *L. pneumophila* within complex biofilms (40). In order to better understand the development of *L. pneumophila* in the environment, this bacterium needs to be examined in complex biofilms.

Iron is an essential nutrient for *L. pneumophila* because its growth depends on the presence of iron in its culture medium (34, 35, 38). Iron also plays a key role in pathogenesis (6, 14) and several iron acquisition pathways have been described for *L. pneumophila*, such as the ferric iron pathway involving the *lbtA/B/C/U* genes (1, 4, 5, 24), the ferrous iron pathway involving the *feoA/B* genes (36), the *frgA* gene in siderophore production (1, 16, 24), and the *iraA/B* genes encoding proteins with the ability to transport iron-loaded peptides in the cytoplasm (42). Iron metabolism in *L. pneumophila* has been reviewed in detail (6). In a previous study, we indicated that two genes, *pvcA* and *pvcB*, which appeared to be related to iron metabolism, were induced in *L. pneumophila* biofilms (17). Furthermore, the addition of iron pyrophosphate at high concentrations was detrimental to biofilm formation by *L. pneumophila* (17). Taken together, these findings suggest that iron is important for biofilm formation by *L. pneumophila*. This is also the case for other bacteria; however, iron was found to be either detrimental or beneficial, similar to *P. aeruginosa*, a model bacteria for biofilm formation (29, 30). Although this is not unexpected because iron is a key nutrient for bacteria, when in excess, iron may lead to the production of toxic compounds such as reactive oxygen species (ROS); therefore, iron homeostasis needs to be tightly controlled (2, 23).

The aim of the present study was to investigate the role of iron in the presence of *L. pneumophila* in complex biofilms. A model of complex biofilms was established with river water. *L. pneumophila* was spiked in river water supplemented with iron pyrophosphate or iron chelators. Biofilm formation was monitored, mainly using qPCR, and the structure of the bacterial population was assessed by T-RFLP.

## Materials and Methods

### Bacterial strains and growth conditions

*L. pneumophila* 130b strain ATCC BAA-74 (also known as Wadsworth or AA100) and six *L. pneumophila* 130b mutants, deficient in iron acquisition, were used in this study: NU269 (*ΔfeoB*) (36), NU229 (*ΔfrgA*) (16), NU244 (*ΔiraB*) (42), NU302 (*ΔlbtA*) (1), NU383 (*ΔlbtU*) (4), and NU311 (*ΔlbtA;frgA*) (24) (Table 1).

*L. pneumophila* strains were cultured in filter-sterilized Buffered Yeast Extract (BYE, 5 g L^−1^ ACES [*N*-(2-Acetamido)-2-aminoethanesulfonic acid, *N*-(Carbamoylmethyl)taurine], 10 g L^−1^ yeast extract; pH 6.9) supplemented with l-cysteine at 0.4 g L^−1^ and iron pyrophosphate at 0.25 g L^−1^, at 37°C under agitation at 150–200 rpm. Solid medium BCYE was obtained by adding charcoal (2 g L^−1^) and agar (15 g L^−1^) to non-filtered BYE. The resulting medium was autoclaved at 121°C for 15 min. Medium was then supplemented with l-cysteine and iron pyrophosphate as described above. When required, antibiotics were used at the following final concentrations: kanamycin at 25 μg mL^−1^ and gentamicin at 2.5 μg mL^−1^. In order to select the chelator concentrations used in this study, *L. pneumophila* was inoculated in BYE not supplemented with iron. When growth reached the middle of the exponential phase, different concentrations of chelators were tested in order to obtain the minimum inhibitory concentration (MIC). Growth was followed by measuring optical density (OD) at 600 nm.

### Biofilm formation

Complex biofilms were formed with natural river water sampled in the Vienne River (sampling location 47°12′45″ N, 0°4′31″ E) in France. Biofilms were allowed to form on polystyrene 12-well microtiter plates (Nunclon Microwell Plates, Nunc) at 37°C for 28 d. The incubation temperature was selected to mimic hot water systems that may be infected by *L. pneumophila*. Water was renewed every two d. During the first 14 d of cultivation, only river water was used for biofilm formation. River water was then spiked with *L. pneumophila* at 10^6^ CFU mL^−1^, supplemented or not with ferric iron pyrophosphate at 335 μmol L^−1^ (corresponding to the concentration found in BYE, *i.e.* 0.25 g L^−1^), deferoxamine mesylate at 20 μmol L^−1^ (DFX: Ferric iron chelator), or 2,2′-dipyridyl at 100 μmol L^−1^ (DIP: Ferrous iron chelator), and then left for two d in the plates. During the last 14 d, river water without *L. pneumophila*, but supplemented or not with ferric iron pyrophosphate (335 μmol L^−1^), DFX (20 μmol L^−1^), or DIP (100 μmol L^−1^), was renewed every two d.

### Isolation of genomic DNA from biofilms

After biofilm formation, the wells in the microtiter plates were washed three times with sterile water to remove non-adherent cells and particles. Biofilms were then scraped off and collected in 1 mL of sterile distilled water. DNA was extracted from biofilm suspensions using a commercial kit (High Pure PCR Template Preparation Kit, Roche) following the manufacturer’s instructions.

### Quantitative PCR

Quantitative PCR assays were performed using the LightCycler FastStart DNA Master^PLUS^ Sybr Green I mix (Roche Applied Science) with a LightCycler 1.5 apparatus (Roche Applied Science). The concentration of total bacteria was analyzed by quantifying the 16S ribosomal RNA gene using the primers 515F (5′-GTGBCAGC MGCCGCGGTAA-3′) and 786R (5′-CTACCAGGGTATCTAATC-3′) (19). Specific primers for *L. pneumophila* were Mip A1 (5′-GCATTGGTGCCGATTTGG-3′) and Mip A2 (5′-GYTTTGC CATCAAATCTTTCTGAA-3′) (43). Free-living amoebae were detected by quantifying the 18S ribosomal RNA gene using two primer couples as previously described (22). The PCR mixture contained 2 μL of sample DNA, 0.5 μL of each primer (final concentration, 0.5 μmol L^−1^), 2 μl of Master Mix and PCR-grade sterile water to a final volume of 10 μL. The run of quantification started with an initial denaturation at 95°C for 10 min, followed by 45 cycles of repeated denaturation (at 95°C for 10 s), annealing (at 60°C for 10 s), and polymerization (at 72°C for 10 s–15 s). A positive control (genomic DNA) and negative control (purified PCR-grade water) were included in all PCR assays. A standard curve was obtained with 10-fold serial dilutions of a known amount of *L. pneumophila* or *Acanthamoeba* genomic DNA. All results are expressed in Genome Units (GU).

### Microscopy

Biofilm formation was followed with the fluorescence microscope Axio observer A1 (Zeiss) using a filter specific for Syto 9 (Zeiss Filter Set 44), a 32x objective (Zeiss). Images were taken using Axiovision Software and processed with the Software Imagis 7.4.1. In order to stain biofilms, wells were washed with sterile water to remove floating particles and planktonic cells. Syto 9 Green Fluorescent Nucleic Acid Stain 6.7 μmol L^−1^ (Molecular Probes, Invitrogen) mixed with Citifluor AF1 (Biovalley) was used to stain microorganisms in biofilms. Biofilms were observed directly in wells.

### Terminal restriction fragment length polymorphism (T-RFLP)

The diversity of the bacterial community in biofilms was analyzed by T-RFLP of the 16S rRNA gene. The primers 8F-FAM (FAM-5′-AGAGTTTGATCMTGGCTCAG-3′), labeled at the 5′-end with 6-carboxyfluorescein (6-FAM), and 1492R (5′-TACGGHTACCTTGTTACGACT-3′) were used to amplify a 1,500 bp fragment of the 16S rRNA gene (25). PCR reactions were performed in 50 μL reaction mixture containing 1×PCR Buffer, deoxynucleoside triphosphate at a final concentration of 0.2 mmol L^−1^ for each, MgCl_2_ at 1.5 mmol L^−1^, the primer at a final concentration of 1 μmol L^−1^ for each, Go Taq DNA polymerase at 1.25 U (Promega, USA), and 2 μL of a 0.5 ng μL^−1^ DNA diluted template. DNA amplification was performed with the vapoprotect thermocycler (Eppendorf), using the following program: initial denaturation (at 95°C for 2 min), followed by 30 cycles consisting of denaturation (at 95°C for 45 s), annealing (at 58°C for 45 s), extension (at 72°C for 1 min), and a final extension (at 72°C for 10 min). Amplified DNA was checked by electrophoresis in 1% agarose gel in 0.5×TBE (Tris-Borate-EDTA).

Fluorescently labeled PCR products (200 μL) were purified using NucleoSpin Gel and PCR Clean-up (Macherey-Nagel) and were eluted at a final volume of 30 μL. Eighty nanograms of fluorescently labeled PCR products were digested with 2.5 U of *Hae*III (Promega) at 37°C for 3 h. T-RFs (terminal restriction fragments) from amplified rRNA products were separated by electrophoresis with a model 3130 automated sequencer (Applied Biosystems) as follows. Each *Hae*III-digested PCR product was mixed with 0.25 μL of GeneScan 500 ROX Size Standard (Applied Biosystems) and 18.75 μL of deionized formamide (Hi-Di Formamide). The sizes of the DNA fragments in the standard were 35, 50, 75, 100, 139, 150, 160, 200, 250, 300, 340, 350, 400, 450, 490, and 500 bases. Each fluorescently labeled *Hae*III-digested PCR product was incubated at 95°C for 3 min, chilled on ice prior to electrophoresis, and resolved by capillary electrophoresis in an ABI 3130 genetic analyzer (Applied Biosystems). T-RFLP electropherograms were analyzed using StatFingerprints Version 2 software (28). All T-RFLP profiles were aligned to the internal standard and to a common baseline. Profiles from 60 to 480 bp were normalized with the minimum value equal to 0.

Data obtained from StatFingerprints software were converted into a data frame subjected to a Principal Component Analysis (PCA), providing the ordination of bacterial communities in a factorial map based on the scores of the first two principal components. Statistical ellipses representing 90% confidence were drawn over the replicates. PCA was performed using ADE-4, R software (33, 41).

### Statistical analysis

qPCR data were analyzed quantitatively by the Mann-Whitney U test, comparing treatments to control conditions, using GraphPad Prism version 6.00 software. Data obtained from StatFingerprints software were converted into a data frame subjected to PCA, providing the ordination of bacterial communities in a factorial map based on the scores of the first two principal components. Statistical ellipses representing 95% confidence were drawn over the replicates. PCA were performed using ADE-4, a R software (33, 41).

## Results

### Time course of biofilm formation

**Fig. 1:**
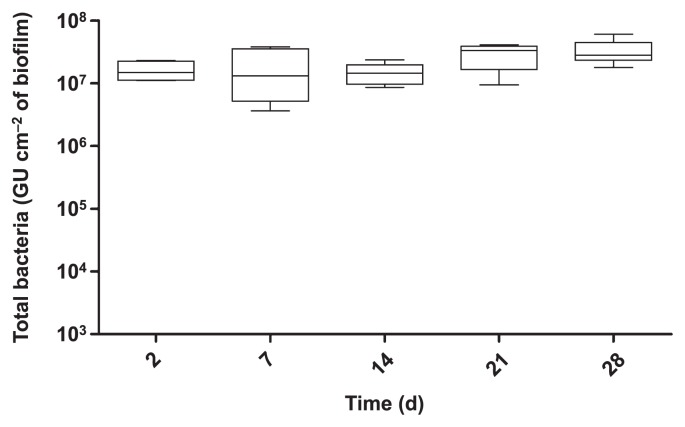
Quantification of total bacteria in the biofilm over 28 d. Data represent the mean±standard error of the mean (SEM) from triplicates of two independent experiments (Mann-Whitney U test).

The time course of biofilm formation was followed in our experimental model of complex biofilms. Biofilms were harvested at different time points and total bacteria were quantified by qPCR using 16S rRNA primers. Biofilms were already established after the first two d, with 10^7^ GU cm^−2^ of the biofilm (Fig. 1). Between 2 and 28 d, the bacterial concentration in the biofilm ranged between 10^7^ and 10^8^ GU cm^−2^ of the biofilm. Therefore, the maximum concentration was rapidly reached and was maintained over the time course of the experiment.

**Fig. 2:**
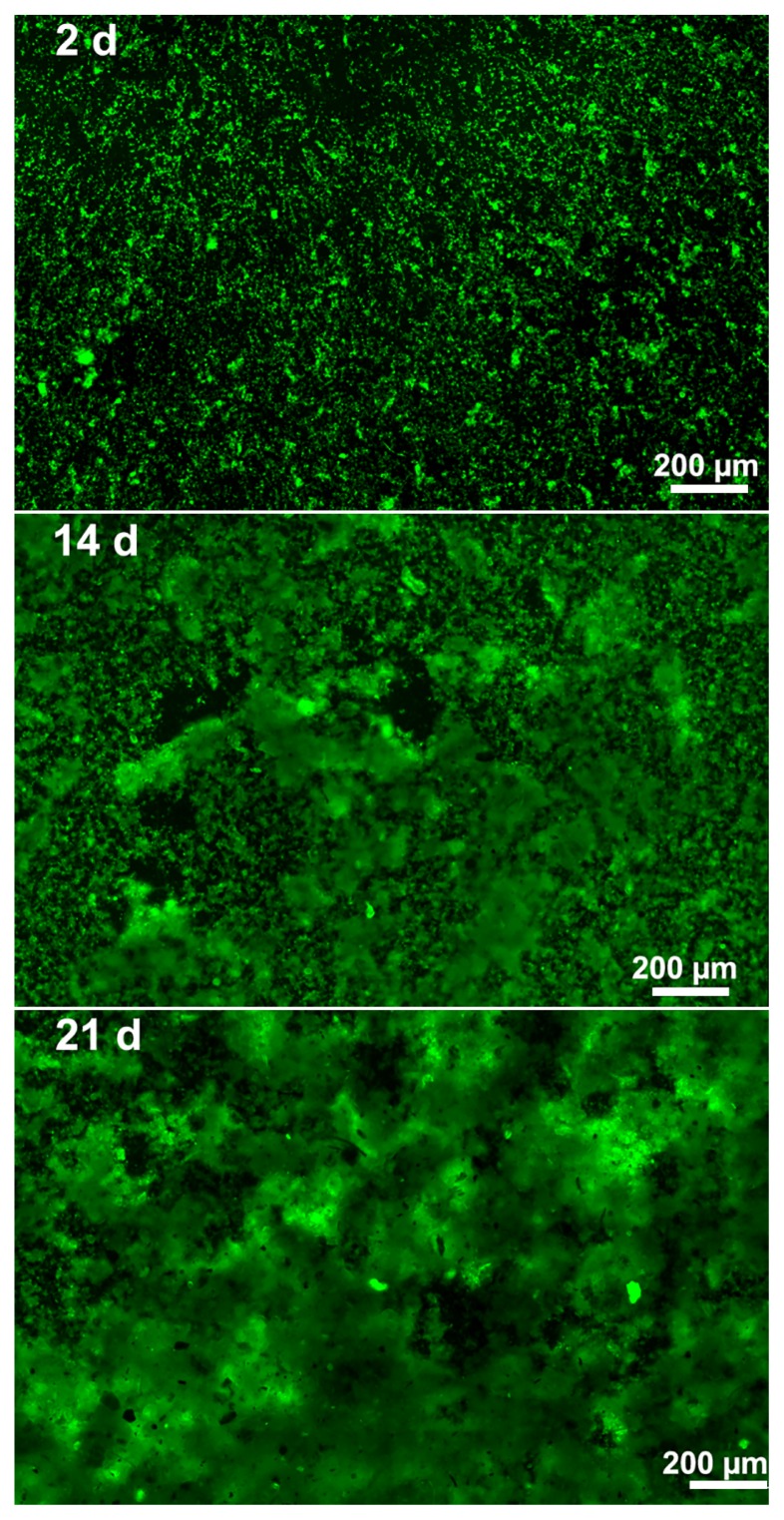
Development of biofilm morphology over 21 d. At different time points (2 d, 14 d, 21 d), biofilms were stained with Syto 9 and observed using fluorescence microscopy (bar size: 200 μm).

Microscopic experiments were performed to follow biofilm establishment and morphology after 2, 14, 21, and 28 d. The results obtained confirmed the establishment of biofilms as early as 2 d after the start of the incubation (Fig. 2). At 14 d and 21 d, there was an increase in the complexity of the architecture and in the thickness of the biofilms (Fig. 2). At 28 d, biofilms were too thick to take sharp images. Therefore, the bacterial number appeared to remain stable, while the biofilm structure was modified over the course of biofilm formation.

### Impact of iron chelator supplementation on *L. pneumophila* growth

**Fig. 3:**
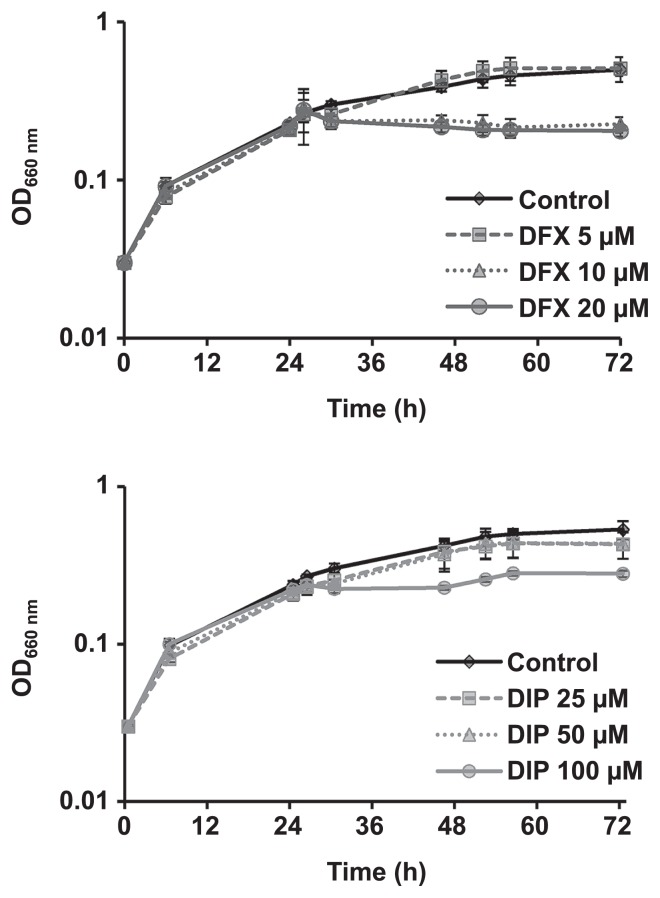
Effects of DFX and DIP on *L. pneumophila* 130b growth. When growth reached OD_600 nm_ 0.7–0.8, culture media were supplemented with different iron chelators at various concentrations: (A) DFX (0, 5, 10, 20 μmol L^−1^) and (B) DIP (0, 25, 50, 100 μmol L^−1^) in order to define their minimal inhibitor concentration (MIC). Data represent the mean±standard deviation (SD) from triplicates of three independent experiments.

In order to follow the impact of iron chelators on *L. pneumophila* growth, various concentrations, which have been reported in the literature, were tested. The growth of our model strain, *L. pneumophila* 130b, was inhibited by DFX, which preferentially chelates ferric iron at 10 and 20 μmol L^−1 (^Fig. 3A), and by DIP, which preferentially chelates ferrous iron at 100 μmol L^−1^ (Fig. 3B). These concentrations, corresponding to MICs in BYE medium, were used in subsequent experiments, which were performed on complex biofilms.

### Role of iron availability on the persistence of *L. pneumophila* in complex biofilms

In order to elucidate the role of iron in the persistence of *L. pneumophila* in biofilms, biofilm formation was conducted with the wild-type *L. pneumophila* 130b strain and various mutant strains with altered iron acquisition (*i.e. ΔfrgA*, *ΔiraB*, *ΔfeoB*, *ΔlbtA*, *ΔlbtA/frgA*, and *ΔlbtU*). Iron availability was modulated by supplementing river water or not with iron pyrophosphate, DFX, or DIP. *L. pneumophila* and total bacteria were quantified by qPCR.

**Fig. 4:**
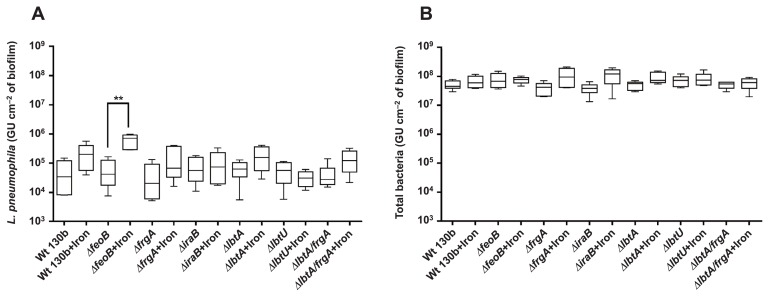
Effects of iron pyrophosphate on the persistence of *L. pneumophila* (A) and total bacteria (B). Results are expressed in GU (Genome Units). Data represent the mean±SEM from triplicates of two independent experiments (**p<0.005 Mann-Whitney U test).

The concentration of iron in river water was approximately 4 μmol L^−1^. Iron supplementation at 335 μmol L^−1^, corresponding to its concentration in BCYE culture medium, did not significantly affect the persistence of *L. pneumophila* in biofilms in any of the strains tested, except for the *feoB* mutant, the quantity of which increased by approximately 1 log from the control condition (Fig. 4A). The values obtained ranged between 10^5^ GU cm^−2^ and 10^6^ GU cm^−2^ of the biofilm. The addition of iron pyrophosphate did not have a significant effect on the quantity of total bacteria (Fig. 4B).

**Fig. 5:**
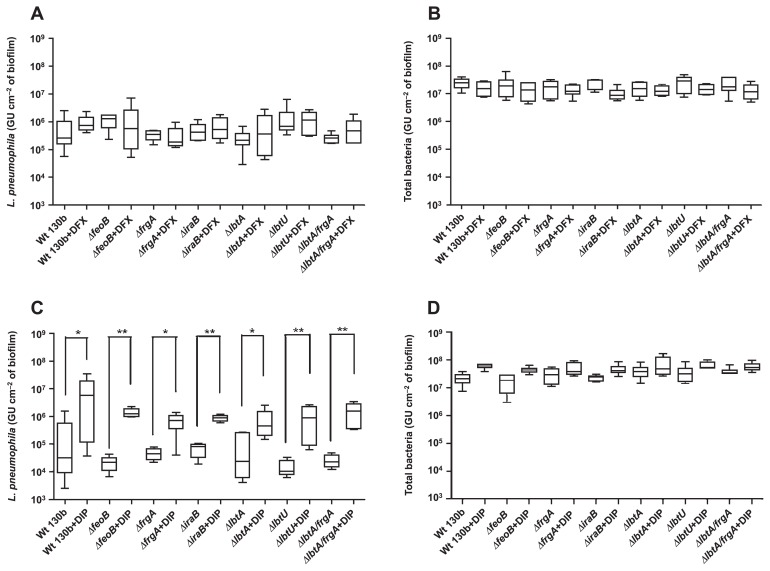
Effects of DFX (20 μmol L^−1^) (A–B) and DIP (100 μmol L^−1^) (C–D) on the persistence of *L. pneumophila* (A–C) and total bacteria (B–D). Results are expressed in GU (Genome Units). Data represent the mean±SEM from triplicates of two independent experiments (*p<0.05; **p<0.005 Mann-Whitney U test).

The addition of DFX did not have a significant effect on the persistence of *L. pneumophila* (WT and mutants) in biofilms (Fig. 5A). In contrast, the addition of DIP promoted the persistence of *L. pneumophila* in biofilms: *L. pneumophila* concentrations in biofilms increased significantly for all strains by 1 to 2 log (Fig. 5C). DFX and DIP had no significant effect on the quantity of total bacteria (Fig 5B–D). These results suggest that DIP had a positive effect on the persistence of *L. pneumophila* independently of mutations in iron acquisition genes.

### Time course of *L. pneumophila* establishment in the presence of DIP

**Fig. 6:**
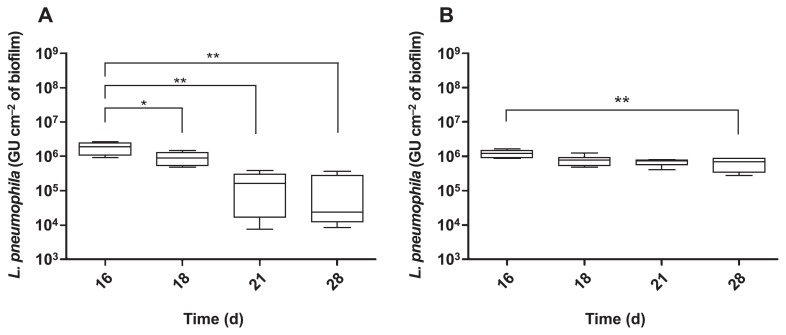
Kinetics of the establishment of *L. pneumophila* in biofilms with (B) or without DIP (100 μmol L^−1^) (A). Results are expressed in GU (Genome Units). Data represent the mean±SEM from triplicates of two independent experiments (*p<0.05; **p<0.005 Mann-Whitney U test).

In order to further elucidate the role of DIP, the time course of the establishment of *L. pneumophila* in biofilms was performed. *L. pneumophila*, quantified by qPCR in biofilms formed without DIP, peaked at approximately 10^6^ GU cm^−2^ of the biofilm (Fig. 6A). This concentration decreased during the course of the experiment. However, this decrease was negligible in biofilms formed with DIP (Fig. 6B) because the concentration of *L. pneumophila* remained constant for 14 d after the addition of bacteria. The addition of DIP appears to contribute, either directly or indirectly, to the persistence of *L. pneumophila* in these complex biofilms.

### Role of free-living amoebae

**Fig. 7:**
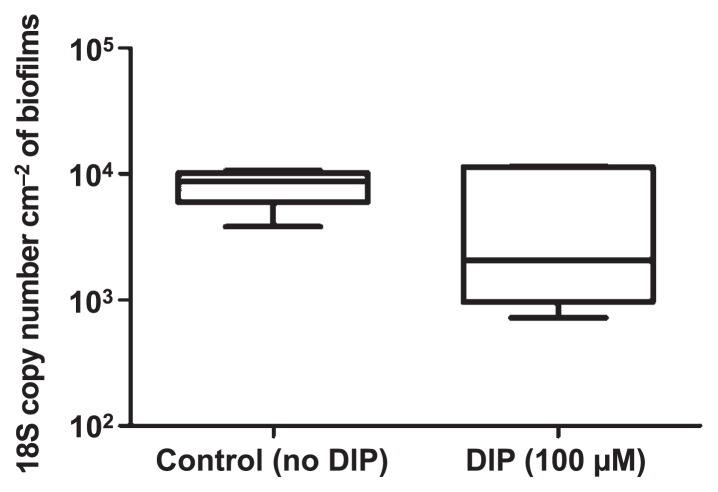
Impact of DIP on amoeba concentrations in biofilms. Results represent the 18S ribosomal RNA copy number. Data represent the mean±SEM from triplicates of two independent experiments (*p*>0.05 Mann-Whitney U test).

Since free-living amoebae are the main reservoir for *L. pneumophila* in water, the impact of DIP on the persistence of free-living amoebae in biofilm was also tested. The quantification of free-living amoebae by qPCR in 28-d-old biofilms revealed that free-living amoebae were present in biofilms, and also that DIP did not significantly affect the amoeba concentration (Fig. 7).

### Impact of iron on bacterial community structures in biofilms

**Fig. 8:**
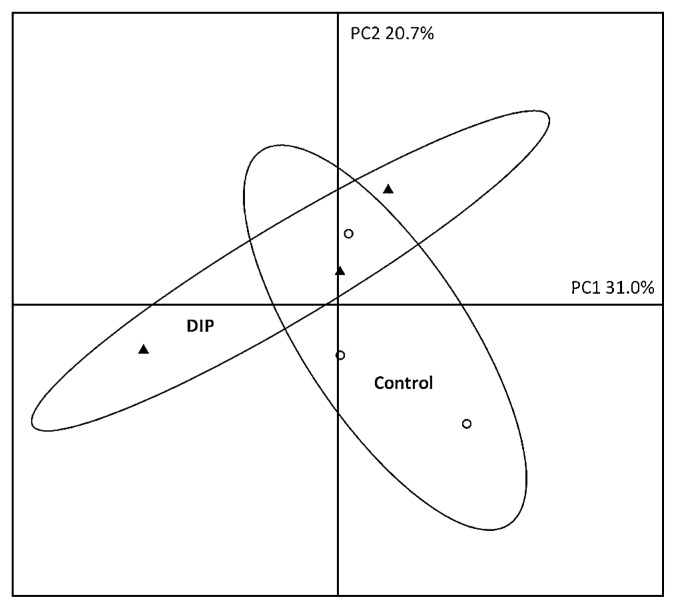
Study of the bacterial community structure with a principal component (PC1 and PC2) factorial map generated from T-RFLP of the 16S rRNA gene. Biofilms were grown for 28 d. At 14 d, *L. pneumophila* 130b was added. River water was supplemented or not (○) with DIP (100 μmol L^−1^) (▲) for the next 14 d. Data represent results from triplicate wells of one single representative experiment. Three repeat experiments gave similar results. Statistical ellipses drawn over the plot of three replicates represent 95% confidence. The percentages of explained variance for the first two principal components are indicated in each ordination.

Since iron availability may modify bacterial community structures, a T-RFLP analysis was performed to compare bacterial populations colonizing the biofilms under control conditions or in the presence of DIP. The PCA of T-RFLP profiles did not reveal any significant discrimination between bacterial communities within the different biofilms (Fig. 8). The statistical ellipses indicating 95% confidence intervals drawn over the replicates of each bacterial biofilm overlapped along PC1 and PC2. Low percentages of variance explaining PC1 and PC2 were obtained. The structure of the bacterial community within biofilms was not significantly affected by the different treatments. This result suggests that DIP did not have a significant impact on the bacterial community structure.

## Discussion

*L. pneumophila* is a human pathogen found in water biofilms. Limited information is available on the parameters that drive *L. pneumophila* persistence in natural biofilms; however, the presence of free-living amoebae and temperatures ranging between 30 and 45°C are clearly important for their growth (40). Since iron is an essential nutrient, it may also have an impact on the presence of *L. pneumophila* in biofilms. In our previous study, we showed that an excess of iron was detrimental to biofilm formation by *L. pneumophila* in a monospecies model (17). *L. pneumophila* is a minor species in natural biofilms; therefore, its behavior needs to be examined in complex biofilms.

In the present study, a complex biofilm model was developed using river water spiked with *L. pneumophila* to form biofilms on microtiter plates. Total bacteria quantification, by qPCR, showed that bacterial numbers were stable and reproducible for the 28 d of the experiment. However, microscopic observations revealed an increase in the complexity of the architecture and in the thickness of the biofilms. This may be explained by the maturation of the biofilm, during which bacteria produce extracellular polymeric substances (EPS) (13).

*L. pneumophila* 130b and various isogenic mutants were tested for their persistence in biofilms with various iron availabilities. The main differences were found with the addition of DIP (ferrous chelator), which increased *L. pneumophila* concentrations in biofilms (Fig. 5C), regardless of the *L. pneumophila* strain (wild-type and mutant strains). These results demonstrated that the effects of DIP were independent of the iron acquisition system because the mutant strains are impaired for iron acquisition. Furthermore, DFX did not have any effect (Fig. 5A), suggesting that only ferrous iron chelation was responsible for this increase. These results were unexpected because chelators were expected to prevent *L. pneumophila* from using iron, and, thus, have a negative effect on its presence in biofilms. Therefore, we hypothesize that the addition of DIP induced the growth of *L. pneumophila* in the biofilms either by modifying the microbial population or protecting *L. pneumophila* against the adverse effects of iron. In order to test this hypothesis, *L. pneumophila* concentrations in the biofilms were followed during the 14 d following the spike and showed that the number of *L. pneumophila* decreased over time in the absence of DIP (Fig. 6A). Otherwise, the presence of DIP clearly stabilized the concentration of *L. pneumophila* over the course of the experiment (Fig. 6B), indicating that DIP actually favored the persistence of *L. pneumophila* in biofilms. The presence of *L. pneumophila* in biofilms is modulated by the occurrence of amoebae or other bacteria (40). In order to clarify whether the bacterial population structure was changed by the addition of chelators or iron pyrophosphate, a T-RFLP experiment in the presence of DIP was performed. No significant difference was observed between the bacterial communities of the different biofilms (Fig. 8), suggesting that the population structure was not markedly affected. It is still possible that discrete modifications occur in biofilms that have an impact on *L. pneumophila* growth. Furthermore, there was no significant modification of the number of amoebae in the presence of DIP (Fig. 7). This result suggests that the effects of DIP were not linked to major changes in the microbial population in the biofilms.

Collectively, the results of the present study suggest that ferrous iron has an impact on the persistence of *L. pneumophila* in biofilms, and this is independent of iron transport genes. These results prompted us to hypothesize that DIP, by chelating ferrous iron, protects *L. pneumophila* against adverse effects due to a decrease in ROS production. A previous study reported that ferrous iron is involved in the Fenton reaction that lead to production of ROS (23), which have antimicrobial activity. Biofilm formation was only affected in the *feoB* mutant, impaired in ferrous iron transport (36), which supports this hypothesis. In bacteria, iron homeostasis is tightly controlled (2) and a critical level of intracellular iron serves as a signal for biofilm development (3). Depending on its concentration, iron may have either a detrimental or beneficial effect on the presence of bacteria in biofilms (29, 30). Each bacterial species has its own pathways to obtain iron for low concentration environments and protect itself against excess iron. In future studies, it will be interesting to assess ROS production and its impact on *L. pneumophila* in the presence of ferrous iron.

## Conclusion

The persistence of *L. pneumophila* in our complex biofilm model was modulated in the presence of DIP, a ferrous iron chelator. Our results suggest that ferrous iron is a key molecule for *L. pneumophila* survival in biofilms. Further studies on the behavior of *L. pneumophila* in complex biofilms are needed in order to obtain a better understanding of the main forces driving its growth in natural biofilms.

## Figures and Tables

**Table 1:** *L. pneumophila* mutants used in this study

Name	Mutated gene	Role of the protein	References
NU269	*ΔfeoB*	Inner membrane transporter of ferrous iron to the cytoplasm	(36)
NU229	*ΔfrgA*	Intracellular protein involved in the production of siderophores chelating Fe^3+^	(1, 16)
NU244	*ΔiraB*	Inner membrane transporter of iron-loaded peptides to the cytoplasm	(42)
NU302	*ΔlbtA*	Intracellular protein involved in legiobactin production	(1)
NU383	*ΔlbtU*	Outer membrane receptor of the legiobactin-Fe^3+^complex	(4)
NU311	*ΔlbtA/frgA*	Intracellular proteins involved in siderophore production	(24)
